# Supplementary material for: Hybrid Models and Biological Model Reduction with PyDSTool
Source: PLoS Comput Biol. 2012 Aug 9;8(8):e1002628. doi: 10.1371/journal.pcbi.1002628 (PMC3415397; doi:10.1371/journal.pcbi.1002628)
Supplement: Text S4 — Complete source code for the PyDSTool package (version 0.88.120504). Includes API documentation and help files linking to web pages. This file is identical to the current public release on Sourceforge.net. (ZIP) [file pcbi.1002628.s004.zip › PyDSTool/html/PyDSTool.Toolbox.FSM-pysrc.html]

xml version="1.0" encoding="ascii"?


PyDSTool.Toolbox.FSM


| Home | Trees | Indices | Help | PyDSTool |
| --- | --- | --- | --- | --- |

Package PyDSTool ::
Package Toolbox ::
Module FSM

[hide private]

[frames] | no frames]

# Source Code for Module PyDSTool.Toolbox.FSM

```
  1  """ 
  2  Finite State Machines. 
  3   
  4  By Noah Spurrier (2002) and other contributors, 
  5  from http://code.activestate.com/recipes/146262-finite-state-machine-fsm/ 
  6   
  7  This module implements a Finite State Machine (FSM). In addition to state 
  8  this FSM also maintains a user defined "memory". So this FSM can be used as a 
  9  Push-down Automata (PDA) since a PDA is a FSM + memory. 
 10   
 11  The following describes how the FSM works, but you will probably also need to 
 12  see the example function to understand how the FSM is used in practice. 
 13   
 14  You define an FSM by building tables of transitions. For a given input symbol 
 15  the process() method uses these tables to decide what action to call and what 
 16  the next state will be. The FSM has a table of transitions that associate: 
 17   
 18          (input_symbol, current_state) --> (action, next_state) 
 19   
 20  Where "action" is a function you define. The symbols and states can be any 
 21  objects. You use the add_transition() and add_transition_list() methods to add 
 22  to the transition table. The FSM also has a table of transitions that 
 23  associate: 
 24   
 25          (current_state) --> (action, next_state) 
 26   
 27  You use the add_transition_any() method to add to this transition table. The 
 28  FSM also has one default transition that is not associated with any specific 
 29  input_symbol or state. You use the set_default_transition() method to set the 
 30  default transition. 
 31   
 32  When an action function is called it is passed a reference to the FSM. The 
 33  action function may then access attributes of the FSM such as input_symbol, 
 34  current_state, or "memory". The "memory" attribute can be any object that you 
 35  want to pass along to the action functions. It is not used by the FSM itself. 
 36  For parsing you would typically pass a list to be used as a stack. 
 37   
 38  The processing sequence is as follows. The process() method is given an 
 39  input_symbol to process. The FSM will search the table of transitions that 
 40  associate: 
 41   
 42          (input_symbol, current_state) --> (action, next_state) 
 43   
 44  If the pair (input_symbol, current_state) is found then process() will call the 
 45  associated action function and then set the current state to the next_state. 
 46   
 47  If the FSM cannot find a match for (input_symbol, current_state) it will then 
 48  search the table of transitions that associate: 
 49   
 50          (current_state) --> (action, next_state) 
 51   
 52  If the current_state is found then the process() method will call the 
 53  associated action function and then set the current state to the next_state. 
 54  Notice that this table lacks an input_symbol. It lets you define transitions 
 55  for a current_state and ANY input_symbol. Hence, it is called the "any" table. 
 56  Remember, it is always checked after first searching the table for a specific 
 57  (input_symbol, current_state). 
 58   
 59  For the case where the FSM did not match either of the previous two cases the 
 60  FSM will try to use the default transition. If the default transition is 
 61  defined then the process() method will call the associated action function and 
 62  then set the current state to the next_state. This lets you define a default 
 63  transition as a catch-all case. You can think of it as an exception handler. 
 64  There can be only one default transition. 
 65   
 66  Finally, if none of the previous cases are defined for an input_symbol and 
 67  current_state then the FSM will raise an exception. This may be desirable, but 
 68  you can always prevent this just by defining a default transition. 
 69   
 70  Noah Spurrier 20020822 
 71  """ 
 72   


73 -class ExceptionFSM(Exception):


74   
 75      """This is the FSM Exception class.""" 
 76   


77 -    def __init__(self, value):


78          self.value = value

 79   


80 -    def __str__(self):


81          return `self.value`

 82   


83 -class FSM(object):


84   
 85      """This is a Finite State Machine (FSM). 
 86      """ 
 87   


88 -    def __init__(self, initial_state, memory=None):


89          """This creates the FSM. You set the initial state here. The "memory" 
 90          attribute is any object that you want to pass along to the action 
 91          functions. It is not used by the FSM. For parsing you would typically 
 92          pass a list to be used as a stack. """ 
 93   
 94          # Map (input_symbol, current_state) --> (action, next_state). 
 95          self.state_transitions = {} 
 96          # Map (current_state) --> (action, next_state). 
 97          self.state_transitions_any = {} 
 98          self.default_transition = None 
 99   
100          self.input_symbol = None 
101          self.initial_state = initial_state 
102          self.current_state = self.initial_state 
103          self.next_state = None 
104          #self.action = None 
105          self.memory = memory

106   


107 -    def reset (self):


108   
109          """This sets the current_state to the initial_state and sets 
110          input_symbol to None. The initial state was set by the constructor 
111          __init__(). """ 
112   
113          self.current_state = self.initial_state 
114          self.input_symbol = None

115   


116 -    def add_transition (self, input_symbol, state, action=None, next_state=None):


117   
118          """This adds a transition that associates: 
119   
120                  (input_symbol, current_state) --> (action, next_state) 
121   
122          The action may be set to None in which case the process() method will 
123          ignore the action and only set the next_state. The next_state may be 
124          set to None in which case the current state will be unchanged. 
125   
126          You can also set transitions for a list of symbols by using 
127          add_transition_list(). """ 
128   
129          if next_state is None: 
130              next_state = state 
131          self.state_transitions[(input_symbol, state)] = (action, next_state)

132   


133 -    def add_transition_list (self, list_input_symbols, state, action=None, next_state=None):


134   
135          """This adds the same transition for a list of input symbols. 
136          You can pass a list or a string. Note that it is handy to use 
137          string.digits, string.whitespace, string.letters, etc. to add 
138          transitions that match character classes. 
139   
140          The action may be set to None in which case the process() method will 
141          ignore the action and only set the next_state. The next_state may be 
142          set to None in which case the current state will be unchanged. """ 
143   
144          if next_state is None: 
145              next_state = state 
146          for input_symbol in list_input_symbols: 
147              self.add_transition (input_symbol, state, action, next_state)

148   


149 -    def add_transition_any (self, state, action=None, next_state=None):


150   
151          """This adds a transition that associates: 
152   
153                  (current_state) --> (action, next_state) 
154   
155          That is, any input symbol will match the current state. 
156          The process() method checks the "any" state associations after it first 
157          checks for an exact match of (input_symbol, current_state). 
158   
159          The action may be set to None in which case the process() method will 
160          ignore the action and only set the next_state. The next_state may be 
161          set to None in which case the current state will be unchanged. """ 
162   
163          if next_state is None: 
164              next_state = state 
165          self.state_transitions_any [state] = (action, next_state)

166   


167 -    def set_default_transition (self, action, next_state):


168   
169          """This sets the default transition. This defines an action and 
170          next_state if the FSM cannot find the input symbol and the current 
171          state in the transition list and if the FSM cannot find the 
172          current_state in the transition_any list. This is useful as a final 
173          fall-through state for catching errors and undefined states. 
174   
175          The default transition can be removed by setting the attribute 
176          default_transition to None. """ 
177   
178          self.default_transition = (action, next_state)

179   


180 -    def get_transition (self, input_symbol, state):


181   
182          """This returns (action, next state) given an input_symbol and state. 
183          This does not modify the FSM state, so calling this method has no side 
184          effects. Normally you do not call this method directly. It is called by 
185          process(). 
186   
187          The sequence of steps to check for a defined transition goes from the 
188          most specific to the least specific. 
189   
190          1. Check state_transitions[] that match exactly the tuple, 
191              (input_symbol, state) 
192   
193          2. Check state_transitions_any[] that match (state) 
194              In other words, match a specific state and ANY input_symbol. 
195   
196          3. Check if the default_transition is defined. 
197              This catches any input_symbol and any state. 
198              This is a handler for errors, undefined states, or defaults. 
199   
200          4. No transition was defined. If we get here then raise an exception. 
201          """ 
202   
203          if self.state_transitions.has_key((input_symbol, state)): 
204              return self.state_transitions[(input_symbol, state)] 
205          elif self.state_transitions_any.has_key (state): 
206              return self.state_transitions_any[state] 
207          elif self.default_transition is not None: 
208              return self.default_transition 
209          else: 
210              raise ExceptionFSM ('Transition is undefined: (%s, %s).' % 
211                  (str(input_symbol), str(state)) )

212   


213 -    def process (self, input_symbol):


214   
215          """This is the main method that you call to process input. This may 
216          cause the FSM to change state and call an action. This method calls 
217          get_transition() to find the action and next_state associated with the 
218          input_symbol and current_state. If the action is None then the action 
219          is not called and only the current state is changed. This method 
220          processes one complete input symbol. You can process a list of symbols 
221          (or a string) by calling process_list(). """ 
222   
223          self.input_symbol = input_symbol 
224          (action_fn, self.next_state) = self.get_transition (self.input_symbol, self.current_state) 
225          if action_fn is not None: 
226              ret = action_fn (self) 
227          else: 
228              ret = None 
229          self.current_state = self.next_state 
230          self.next_state = None 
231          return ret

232   
233   


234 -    def process_list (self, input_symbols):


235   
236          """This takes a list and sends each element to process(). The list may 
237          be a string or any iterable object. """ 
238   
239          for s in input_symbols: 
240              self.process (s)

241   
242   


243 -class ObjFSM(FSM):


244      '''A subclass of FSM where input_symbol may be any kind of object, even an unhashable one. 
245      For each input_symbol to process, the machine will try a sequence of functions; 
246      the first to return True determines (action, next_state). 
247      ''' 
248   


249 -    def add_transition(self, test, state, action, next_state):


250          self.state_transitions.setdefault(state, []).append((test, action, next_state))

251   


252 -    def get_transition(self, input_symbol, state):


253          #input_symbol arg is not used, but we keep it for compatibility with FSM class 
254          for (test, action, next_state) in self.state_transitions.get(state, []): 
255              if test(self): 
256                  return (action, next_state) 
257          try: 
258              return self.state_transitions_any[self.current_state] 
259          except KeyError: 
260              pass 
261          if self.default_transition != None: 
262              return self.default_transition 
263          raise ExceptionFSM('Transition is undefined: (%s, %s).' % 
264              (str(input_symbol), str(self.current_state)) )

265
```

  


| Home | Trees | Indices | Help | PyDSTool |
| --- | --- | --- | --- | --- |

|  |  |
| --- | --- |
| Generated by Epydoc 3.0.1 on Fri May 4 15:24:22 2012 | http://epydoc.sourceforge.net |
